# A randomized controlled phase III study comparing hadrontherapy with carbon ions versus conventional radiotherapy – including photon and proton therapy – for the treatment of radioresistant tumors: the ETOILE trial

**DOI:** 10.1186/s12885-022-09564-7

**Published:** 2022-05-23

**Authors:** Jacques Balosso, Olivia Febvey-Combes, Annie Iung, Hélène Lozano, Abdoulkader Soumai Alloh, Catherine Cornu, Magali Hervé, Zohra Akkal, Michel Lièvre, Valérie Plattner, Francesca Valvo, Cristina Bono, Maria Rosaria Fiore, Viviana Vitolo, Barbara Vischioni, Stéphanie Patin, Hubert Allemand, François Gueyffier, Jennifer Margier, Pascale Guerre, Sylvie Chabaud, Roberto Orecchia, Pascal Pommier

**Affiliations:** 1 Centre François Baclesse, Service de radiothérapie, BP 45026, F-14076 Caen, Cedex 5 France; 2 Hospices Civils de Lyon, Pôle de Santé Publique, Lyon, France; 3 Hospices Civils de Lyon, Direction de la Recherche en Santé, Lyon, France; 4 UMR 5558, Université Claude Bernard Lyon 1, Lyon, France; 5 INSERM, CIC1407, Hospices Civils de Lyon, Bron, France; 6 Centro Nazionale di Adroterapia Oncologica, Pavia, Italy; 7 Groupement Coopération Sanitaire Centre Etoile, Lyon, France; 8 Caisse Nationale d’Assurance Maladie des Travailleurs Salariés, Paris, France; 9 Université de Lyon, Université Claude Bernard Lyon 1, P2S UR4129, Lyon, France; 10 Centre Léon Bérard, Direction de la Recherche Clinique et de l’Innovation, Lyon, France; 11 European Institute of Oncology IRCCS, Milan, Italy; 12 Centre Léon Bérard, Service de Radiothérapie, Lyon, France

**Keywords:** Radiotherapy, Carbon ion therapy, Proton therapy, Photon therapy, Radioresistant tumors

## Abstract

**Background:**

Some cancers such as sarcomas (bone and soft tissue sarcomas) and adenoid cystic carcinomas are considered as radioresistant to low linear energy transfer radiation (including photons and protons) and may therefore beneficiate from a carbon ion therapy. Despite encouraging results obtained in phase I/II trials compared to historical data with photons, the spread of carbon ions has been limited mainly because of the absence of randomized medical data. The French health authorities stressed the importance of having randomized data for carbon ion therapy.

**Methods:**

The ETOILE study is a multicenter prospective randomized phase III trial comparing carbon ion therapy to either advanced photon or proton radiotherapy for inoperable or macroscopically incompletely resected (R2) radioresistant cancers including sarcomas and adenoid cystic carcinomas.

In the experimental arm, carbon ion therapy will be performed at the National Center for Oncological Hadrontherapy (CNAO) in Pavia, Italy. In the control arm, photon or proton radiotherapy will be carried out in referent centers in France.

The primary endpoint is progression-free survival (PFS). Secondary endpoints are overall survival and local control, toxicity profile, and quality of life. In addition, a prospective health-economic study and a radiobiological analysis will be conducted.

To demonstrate an absolute improvement in the 5-year PFS rate of 20% in favor of carbon ion therapy, 250 patients have to be included in the study.

**Discussion:**

So far, no clinical study of phase III has demonstrated the superiority of carbon ion therapy compared to conventional radiotherapy, including proton therapy, for the treatment of radioresistant tumors.

**Trial registration:**

ClinicalTrials.gov identifier: NCT02838602. Date of registration: July 20, 2016. The posted information will be updated as needed to reflect protocol amendments and study progress.

**Supplementary Information:**

The online version contains supplementary material available at 10.1186/s12885-022-09564-7.

## Background

Radiotherapy is improving rapidly and is being enhanced by innovative high-technology equipment using hadrontherapy by proton and carbon ion beams to destroy tumours [1, 2]. Compared to photons (X-rays), a beam of heavy charged particles (protons and carbon ions) enables significantly higher ballistic accuracy with the expected therapeutic benefit of an improvement of quality of life and chances of recovery [3].

Inherently mediocre ballistics selectivity of photons can be compensated by the use of a large number of beams (multiplication of entrance portals) and by modulation of the intensity of the beam enabling the dose to be accurately conformed to the target volume, with the main consequence of irradiating a much larger volume of healthy tissue to medium and low doses. This technique is used for innovative methods of photon radiation therapy: intensity-modulated radiation therapy (IMRT) using either advanced linear accelerators or specific tomotherapy devices and, for small volume tumors, stereotactic radiotherapy using either a conventional linear accelerator specially equipped or a dedicated mini-accelerator mounted on a robotic arm (the Cyberknife®).

Carbon ions also have a much higher biological efficiency (cell destruction) at the Bragg peak (and not in the entrance channel) at the same physical dose than so-called low linear energy transfer (LET) radiation, such as photons and protons [4, 5]. This relative biological effectiveness (RBE) is variable depending on the tissues passed through and is markedly higher for radioresistant cancers [6]. This intrinsic radiological property is the main argument for the use of carbon ions compared to protons.

Carbon ion radiotherapy has shown promising results for a variety of malignancies in prospective phase I/II or phase II studies [4, 7]. In particular, the medical benefit of carbon ions is expected to be major for non-operable (or incompletely resected) localized cancers that are radioresistant to low LET (photons and protons) and located near radiosensitive organs at risk. However, high level evidence from phase III studies demonstrating the benefit of carbon ions is not available [5, 8].

Certain types of cancers are known for their intrinsic radioresistance (melanomas, some sarcomas, adenoid cystic carcinomas) and are thus the main indications of carbon ions. Other types of cancers that are more frequent (e.g., squamous cell carcinomas, adenocarcinomas) may have extremely variable radiosensitivity, linked in particular to the existence of tumor hypoxia, but also to intrinsic biological characteristics that are still poorly understood and difficult to prospectively identify.

In the present study, two groups of indications known for their intrinsic radioresistance and for which the medical data obtained in the pioneering centers for carbon ion therapy are the most documented will be considered: sarcomas [9, 10] and adenoid cystic carcinomas of the head and neck [11–14]. Spinal and sacral chordomas and chondrosarcomas are dully included, but similar tumours of the base of the skull are currently the subject of randomized phase III studies comparing protons and carbon ions, and are thus excluded from the indications of the present study [15, 16].

We set up a multicenter randomized phase III study aimed at evaluating the clinical benefit of hadrontherapy based on carbon ions compared to the best modalities of photon radiation therapy or protons in these radioresistant tumors.

## Methods and design

### Study design

The ETOILE trial is a multicenter open-label randomized parallel-group superiority trial comparing carbon ion therapy with standard radiotherapy, including a treatment by photons or protons. Patients will be enrolled in French reference centers for cancer treatment. Patients fulfilling the eligibility criteria will be randomized into two arms using a 1:1 ratio (experimental arm: carbon ion therapy; control arm: photon or proton therapy). Follow-up takes place 2 months and 6 months after the end of radiotherapy, then every 6 months during 5 years.

### Study objectives and endpoints

The primary outcome is the progression-free survival (PFS) defined as the time from randomization to the first relapse or death, whatever the cause, or to the latest news date. Progression is defined according to RECIST v1.1 criteria [17] based on standard medical imaging (CT or MRI), and confirmed 6 months later. An independent committee unaware of the treatment group will validate all progressions.

Secondary outcomes include: the tolerance profile assessed using CTCAE-V4.02 grades; survival without locoregional progression; survival without metastases; overall survival; quality of life using the EQ-5D-3L. Associated health-economic and radiobiological studies are also planned.

### Patient selection

#### Inclusion/randomization criteria

Patients eligible for inclusion in this study have to meet all of the following criteria:

- age ≥ 18 years
- unresectable or inoperable cancer or a cancer with a macroscopically incomplete resection (R2)
- radioresistant cancers including: adenoid cystic carcinomas of the head and neck (excluding laryngeal and tracheal sites), soft tissue sarcomas, rhabdomyosarcoma of pleomorphic type only, retroperitoneal sarcomas providing technical feasibility, osteosarcomas of any localization and whatever the grade (Ewing’s sarcomas excluded), chondrosarcoma (excluding skull base) of WHO grade ≥ 2, chordomas of the axial skeleton and the pelvis (base of the skull excluded), and angiosarcomas (scalp angiosarcoma excluded).
- absence of epidermal invasion (a hypodermic invasion is accepted with cutaneous adherence but no permeation of the epidermis);
- largest dimension of the macroscopic volume (Gross Tumor Volume, GTV) < 20 cm;
- Performance Status ECOG ≤2 or Karnofsky Index ≥60;
- no severe comorbidities and life expectancy over 10 years;
- patient physically and mentally able to accept and receive cares far from their home;
- no ongoing pregnancy or risk of pregnancy for women of childbearing age;
- patient affiliated with a social security insurance;
- written informed consent to participate in the study.

Additional criteria are requested for randomization:

- validation of an indication for radiotherapy by a specialized local multidisciplinary tumor board (MTB) and, in the context of the indications of this study, validation of the indication and the feasibility for carbon ion therapy by the medical team of the National Center for Oncological Hadrontherapy (CNAO) in Pavia, Italy;
- possibility of offering carbon ion therapy within 2 months.

#### Non-inclusion/non-randomization criteria

Patients eligible for this study must not meet any of the following criteria:

- macroscopically or microscopically complete surgical excision (R0 or R1);
- a history of radiotherapy on the site and region to be treated;
- metastatic disease;
- disease not relevant for a curative approach (e.g. a very advanced disease with extensive regional invasion or with accelerated progression resistant to all medical treatments, especially sarcomas);
- contra-indication to the realization of a radiotherapy (by photons, protons or carbon ions) for medical or technical reasons;
- pre-scheduled surgery or chemotherapy after radiotherapy;
- a history or concomitant presence of another cancer; with the exception of an in situ cervical cancer or curatively treated basal cell cutaneous carcinoma or any other curatively treated cancer without signs of a relapse for at least 5 years;
- simultaneous participation in another prospective trial;
- patient unable to be followed-up for 5 years.

### Randomization

Randomization will be done using an Interactive Web Response Systems (IWRS). Stratification parameters include the center and the type of cancer (adenoid cystic carcinoma versus sarcoma and chordoma). As the trial design is open-label, no blinding of treatment assignment is possible.

### Reference committee

An Independent Data Safety Monitoring Board (IDSMB) is established to ensure the protection of patients, to ensure that the study is conducted in an ethical manner, to systematically reassess the benefit-risk balance of the trial and to ensure the independent review of the scientific results under study. The IDSMB will be composed of independent experts in the field of radiation oncology and methodology of clinical trials.

### Radiation therapy

#### Experimental arm: carbon ion therapy

In the carbon ion arm, patients will not receive conventional radiotherapy. For all patients randomized in this treatment arm, carbon therapy will be delivered at the CNAO in Pavia, Italy. The total duration of treatment will be 4 weeks (16 sessions, 4 sessions per week). Patients will receive support from the study team and the local team for their transportation and housing.

#### Control arm: standard photon or proton therapy

Patients will be able to receive two different types of radiotherapy in the control arm (photon or proton therapy). Standard treatment will be delivered in French regional centers for cancer treatment and university hospital centers, some private centers, and proton therapy centers.

Radiotherapy with intensity modulation (IMRT) will be delivered using most advanced procedures in the center recruiting the patients, after one or more preparation sessions (scanner, simulation and implementation under machine, verification by quality control imaging). The IMRT solution will be left to the investigator’s choice (standard IMRT, dynamic arctherapy, tomotherapy) according to local technical availability and the clinical situation. 3D conformal radiation therapy without intensity modulation will also be an option if it allows dosimetric results comparable to those of IMRT on the basis of the resulting dose-volume-histograms (DVH). All recommended technical modalities in terms of contention, repositioning capacity and quality control for image-guided radiation therapy (IGRT) will be implemented. Planned and performed treatment data will be recorded according to the current recommendations of the International Commission on Radiation Units and Measurements (ICRU).

**Fig. 1:**
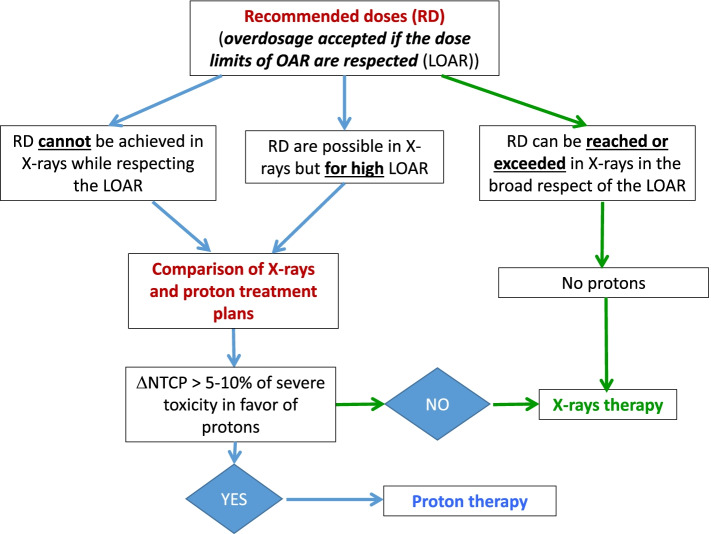
Choice between photon and proton therapy in the control arm. ΔNTCP, Difference in normal tissue complication probability; LOAR, Dose limits of organs at risk; OAR, Organs at risk; RD, Recommended doses

Deep proton therapy will be performed only for some patients based on quantitative criteria for probable differences in normal tissue complication probability (NTCP) calculated by comparing a photon treatment plan with a proton treatment plan. The indication of proton therapy may result from the observation of an excessive toxic risk with photon therapy. Proton therapy can be exclusive or complementary to a photon therapy treatment when a low-risk volume is defined and substantially greater than the high-risk volume. In cases of photon-proton association, the proton part will be carried out first in order to give it the maximum of possibilities to adapt to the anatomical constraints without being disturbed by previously received doses. The use of proton therapy will be decided using an objective approach described in Fig. 1.

This strategy will offer the opportunity to scientifically evaluate the feasibility and relevance of this approach based on NTCP models [18].

#### Dose prescription

In the experimental arm, the carbon ion therapy will be delivered according to the methods of the National Institute of Radiological Sciences (NIRS), Japan, and the Heidelberg Ion-Beam Therapy Center (HIT), Germany, adapted by the CNAO. As an indication, doses of carbon ion therapy to deliver are given in Table 1.

**Table 1:** Experimental arm (carbon ion therapy)

Diseases	Methods	Doses (Gy (IsoE))^b^
Adenoid cystic carcinomas	CNAO^a^	60.8 to 64.0
Sarcomas	CNAO^a^	70.4 to 73.6
Chordomas	CNAO^a^	70.4 to 73.6

^a^adapted from the experience of NIRS, National Institute of Radiological Sciences (Japan) and HIT, Heidelberg Ion-Beam Therapy Center (Germany)

^b^All treatments are delivered in 16 fractions, 4 fractions per week

CNAO, National Center for Oncological Hadrontherapy; IsoE, Isoeffective dose to ^60^Co or Rx doses delivered with 2 Gy per fraction

For patients included in the standard arm (photon or proton therapy), minimum recommended doses are given in Table 2.

**Table 2:**
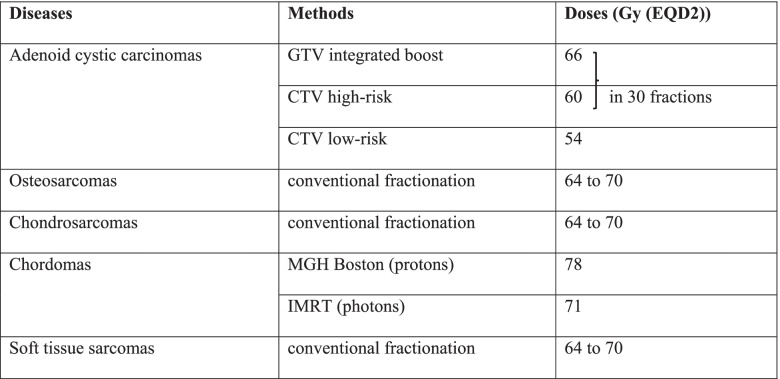
Control arm (standard photon or proton therapy)

*CTV* Clinical target volume, *EQD2* Equivalent dose with 2 Gy per fraction, *GTV* Gross tumor volume, *IMRT* Intensity-modulated radiation therapy, *MGH* Massachusetts General Hospital

These dose recommendations are given for information purposes and are likely to change over time.

### General organization

**Fig. 2:**
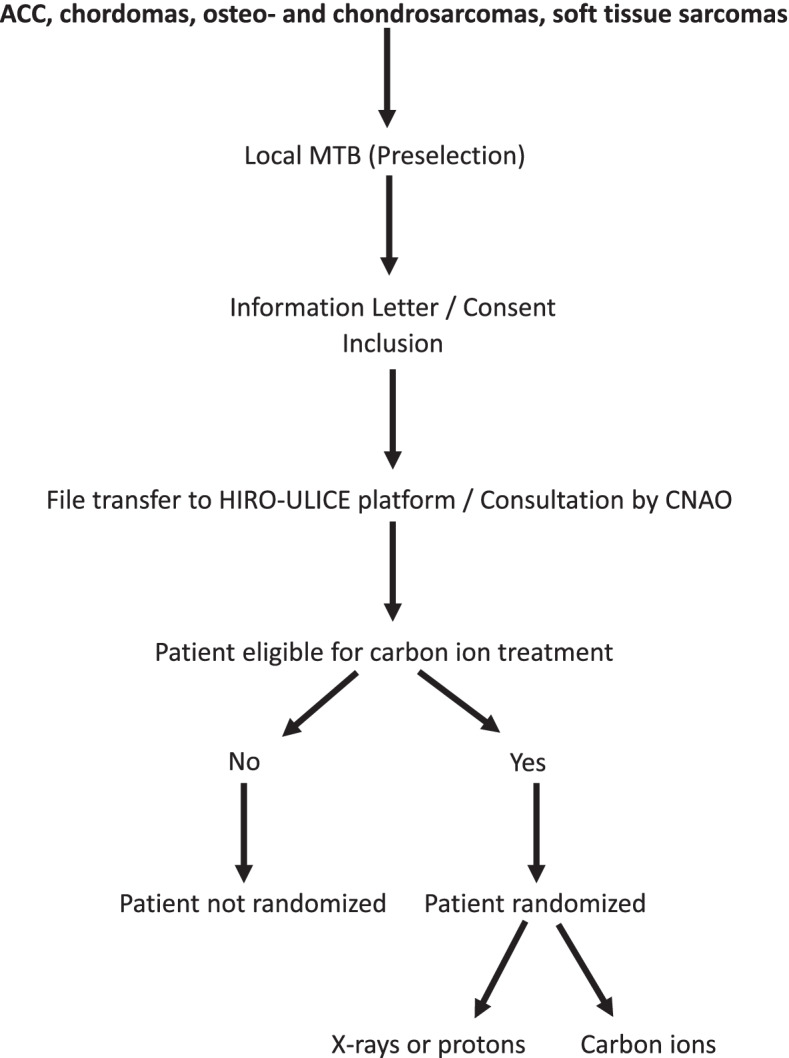
Workflow diagram. ACC, Adenoid cystic carcinomas; CNAO, National Center for Oncological Hadrontherapy; MTB, Multidisciplinary tumor board

A central platform from the Heidelberg Institute for Radiation Oncology (HIRO), the so-called *HIRO-ULICE platform*, will be used for storage and exchange of data such as anonymized medical imaging and patient medical records. This platform will allow the review of patient documents by proton centers for patients in the standard group requiring proton therapy, the confirmation by CNAO that carbon therapy is feasible, and a quality control by the study team. Figure 2 presents the workflow.

The Ennov Clinical trial software (version 8.2.50) will be used for randomization, clinical data collection, and central data management.

### Sample size calculation

The study is calibrated to detect a treatment effect hazard ratio (HR) of 0.515 under the proportional hazards assumption, translating in an absolute improvement of the 5-year PFS rate of 20% in favor of the experimental arm (70% in the experimental arm vs 50% in the control arm).

A total of 92 events [East Software- Copyright@Cytel inc. 1994] notified in the study would have 90% power to show statistically significant PFS at a 2-sided alpha. Considering initial recruitment duration of 24 months and a 5-year follow-up for the last included patient, 108 patients per arm would be randomized in the study. To optimize the study and consider the possibility of withdrawal of consent and patients lost to follow-up, the recruitment objective was set at 250 patients.

### Statistical analysis

#### Primary endpoint

The primary endpoint will be analyzed on the intention-to-treat (ITT) population comprising all randomized patients in the arm where they were randomized. A per-protocol analysis will also be performed in patients without major deviation from the protocol. The evaluable population for treatment tolerance will be all patients who received at least one session of radiotherapy of any kind. PFS will be calculated from the date of randomization to the date of first evidence of a documented relapse, or until the date of death regardless of cause, or until the date of last news (data from patients who did not present the event at the time of the analysis will be censored). It will be estimated in each arm by the Kaplan-Meier method and compared between the two treatment arms by a Log-Rank test.

#### Secondary endpoints

The overall survival analysis will be performed in the ITT population using the same methods.

Tolerance assessments will be conducted throughout treatment and will continue until completion of follow-up using CTCAE-V4.02 criteria. Two periods will be distinguished, events appearing during or within 2 months of the treatment period and late toxicities appearing within 5 years after the end of treatment and which will be considered attributable to radiotherapy. The rate of patients with at least one adverse event will be compared between the two treatment arms.

Quality of life will be evaluated at baseline, at 6 months and then at 1 year and annually until the fifth year of follow-up after treatment using the EQ-5D questionnaire. The quality of life analysis will be carried out on the ITT population. The level of quality of life at inclusion and its evolution during the monitoring period will be compared between the treatment arms. The 10% difference between initial assessment and subsequent assessments of the overall quality of life score will be considered clinically relevant as defined by Osoba et al. [19, 20].

#### Interim analysis

An interim analysis is planned for effectiveness or futility. This analysis will be carried out when approximately 50% (46 events) of the final number of PFS events has been reached. The significance level at this interim analysis to establish the superiority or futility of experimental treatment over control with regard to PFS will be determined based on the observed number of events, using the O’Brien & Fleming boundaries as implemented by the Lan-DeMets alpha spending method (Lan and DeMets 1983) in order to control the overall Type I error rate.

Using planned enrollment rate, this analysis should be performed 42 months after first included patient. For example, if the number of events at the time of interim efficacy analysis is exactly 46, the bounds to use during this analysis according to Lan Demets rules will be *p* = 0.0031 and *p* = 0.8443 respectively to reject H0 and H1.

### Health economic analysis

The main objective is to evaluate the efficiency of a treatment with carbon ions compared to the standard treatments used in the specific indications of the clinical study. We will performed both Cost-Effectiveness Analysis (CEA) and Cost-Utility Analysis (CUA) at 5 years from French public health care system perspective. The endpoints are the incremental cost-effectiveness ratios (ICERs). They are defined by the difference in cost between the two interventions, divided by the difference in effect. The effects will be measured by the overall survival for the CEA and by the quality-adjusted life years (QALY) for the CUA. Utility will be measured with the EQ-5D-3L questionnaire.

Additionally, in this study carbon ion therapy is carried out abroad and invoiced to the French National Health Insurance Fund (CNAM). Under the hypothesis of a newly implanted hadrontherapy center in France, we will have to estimate the cost of this future act of treatment (and its associated preparation act) for the hospital. We will rely on data from the literature (cost studies carried out in France and abroad) and from the Italian and German experts (concerning the average time spent by the various categories of staff for the various stages of processing and its preparation).

### Radiobiological analysis

Initial biopsy samples will be analyzed by large-scale genomic processes to identify genetic tumor profiles correlated with the clinical outcomes. This part of the study will be carried out during the course of the trial.

## Discussion

When indicated, conventional radiotherapy is currently the reference in the treatment of bone and soft tissue sarcomas as well as adenoid cystic carcinomas. High-LET beams such as carbon ions theoretically offer biologic advantages compared to conventional radiotherapy in these indications. The ETOILE study is thus designed as a prospective multicenter randomized phase III trial evaluating the superiority of carbon ion therapy over conventional methods including photons and protons, and will be carried out in France and Italy.

Several factors have limited the spread of carbon ion therapy. First of all, the absence of randomized medical data that would conclusively demonstrate the therapeutic gain provided by carbon ion therapy, linked to a very limited treatment capacity but also to the difficulty in establishing a reference comparator treatment for these rare indications [21].

The opening of the HIT in Germany in 2009 marked a crucial turning point in the establishment of randomized protocols comparing protons and carbon ions for different locations [15, 16, 22–24]. More recently, other randomized trials are planned to be conducted in carbon ion therapy centers worldwide [8, 25–27].

Carbon ion therapy has already been applied to more than 40,000 patients worldwide, including about 50 patients in France. So far, there is no available evidence from randomized phase III studies of the efficacy of carbon ion therapy on cancer outcomes [5, 8]. Moreover, to the best of our knowledge, all advanced phase III trials are carried out as monocentric trials restricting generalizability.

The primary aim of the ETOILE study is to demonstrate whether the biological advantages of carbon ion therapy can be translated as a clinical advantage, and to evaluate the predictive value of molecular markers, for a better orientation of the patients requiring a treatment by carbon ion therapy.

### Status of the trial

The ETOILE study started in December 2017 and patient recruitment is ongoing at March 23, 2022.

## Supplementary Information

**Additional file 1.** ETOILE trial – List of participating centers. Full names of all participating centers.

## Data Availability

Data generated or analyzed during the study will be available from the corresponding author on reasonable request. Results will be communicated via publications in peer-reviewed journals and presentations at international conferences.

## Abbreviations

CNAO: National Center for Oncological Hadrontherapy
CTV: Clinical target volume
GTV: Gross Tumor Volume
HIRO: Heidelberg Institute for Radiation Oncology
HIT: Heidelberg Ion-Beam Therapy Center
IMRT: Intensity-modulated radiation therapy
LET: Linear energy transfer
MTB: Multidisciplinary tumor board
NIRS: National Institute of Radiological Sciences
NTCP: Normal tissue complication probability
PFS: Progression-free survival
