# Supplementary material for: A randomized controlled phase III study comparing hadrontherapy with carbon ions versus conventional radiotherapy – including photon and proton therapy – for the treatment of radioresistant tumors: the ETOILE trial
Source: BMC Cancer. 2022 May 23;22:575. doi: 10.1186/s12885-022-09564-7 (PMC9128242; doi:10.1186/s12885-022-09564-7)
Supplement: Supplementary file 1 — Additional file 1. ETOILE trial – List of participating centers. Full names of all participating centers. [file 12885_2022_9564_MOESM1_ESM.docx]

**Supplementary file ETOILE trial – List of participating centers**

Centre Léon Bérard, Lyon, France

Institut Curie – Centre de Protonthérapie, Orsay, France

CHU Grenoble, Grenoble, France

CHU Pitié-Salpêtrière, Paris, France

Institut de Cancérologie Lucien Neuwirtz, Saint-Priest-en-Jarez, France

Institut de Cancérologie de Lorraine Alexis Vautrin, Vandœuvre-Lès-Nancy, France

Institut Gustave Roussy, Villejuif, France

Centre Georges-François Leclerc, Dijon, France

Centre Jean Perrin, Clermont-Ferrand, France

Centre Paul Strauss, Strasbourg, France

Centre René Gauducheau, Saint-Herblain, France

Centre Oscar Lambret, Lille, France

Centre Antoine Lacassagne, Nice, France

Institut Claudius Regaud – Institut universitaire du cancer de Toulouse Oncopole, Toulouse, France

CHU Bordeaux – Hôpital Haut-Lévêque, Pessac, France

Centre François Baclesse, Caen, France

Institut Régional du Cancer Montpellier – Val d'Aurelle, Montpellier, France

Institut Jean Godinot, Reims, France

CHU Amiens, Amiens, France

Centre Eugène Marquis, Rennes, France

Institut Bergonié, Bordeaux, France

Hôpital de la Timone, Marseille, France
